# Supplementary material for: Molecular Cloning and Functional Analysis of DXS and FPS Genes from Zanthoxylum bungeanum Maxim
Source: Foods. 2022 Jun 14;11(12):1746. doi: 10.3390/foods11121746 (PMC9223008; doi:10.3390/foods11121746)
Supplement: Supplementary file 1 [file foods-11-01746-s001.zip › Figure_S1.pdf]

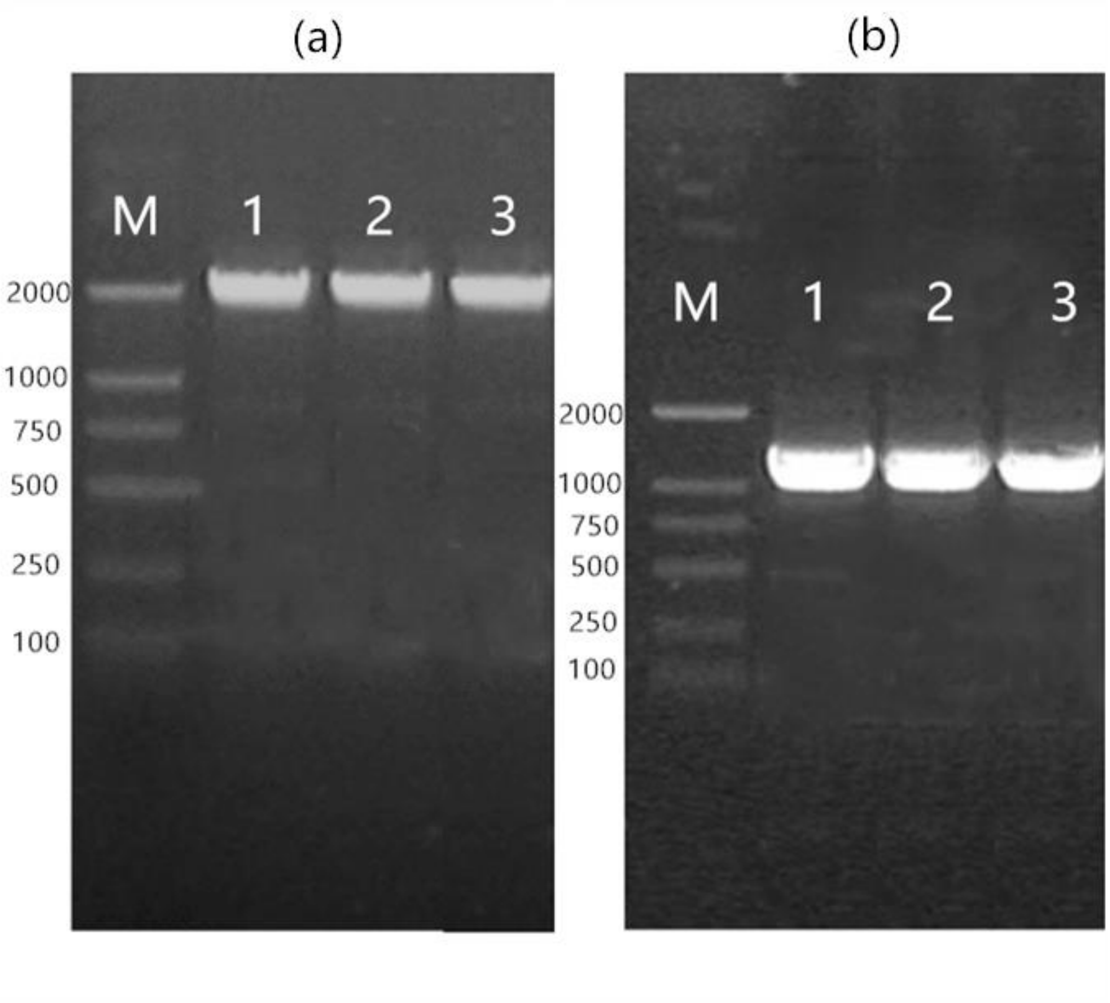

Figure S1. Amplification of CDSs of *ZbDXS* and *ZbFPS* in *Z. bungeanum*. *ZbDXS* amplification. M: DNA marker (DL2000) 1-3: PCR products (a). *ZbFPS* amplification. M: DNA marker (DL2000) 1-3: PCR products (b).
