# Supplementary material for: Molecular Cloning and Functional Analysis of DXS and FPS Genes from Zanthoxylum bungeanum Maxim
Source: Foods. 2022 Jun 14;11(12):1746. doi: 10.3390/foods11121746 (PMC9223008; doi:10.3390/foods11121746)

Figure S2. Deduced amino acid sequences of the conserved regions of ZbDXS. The conserved domains are boxed. Homology level was highlighted by shading in color: black for 100%, pink for ⩾ 75% and blue for ⩾ 50% identity.

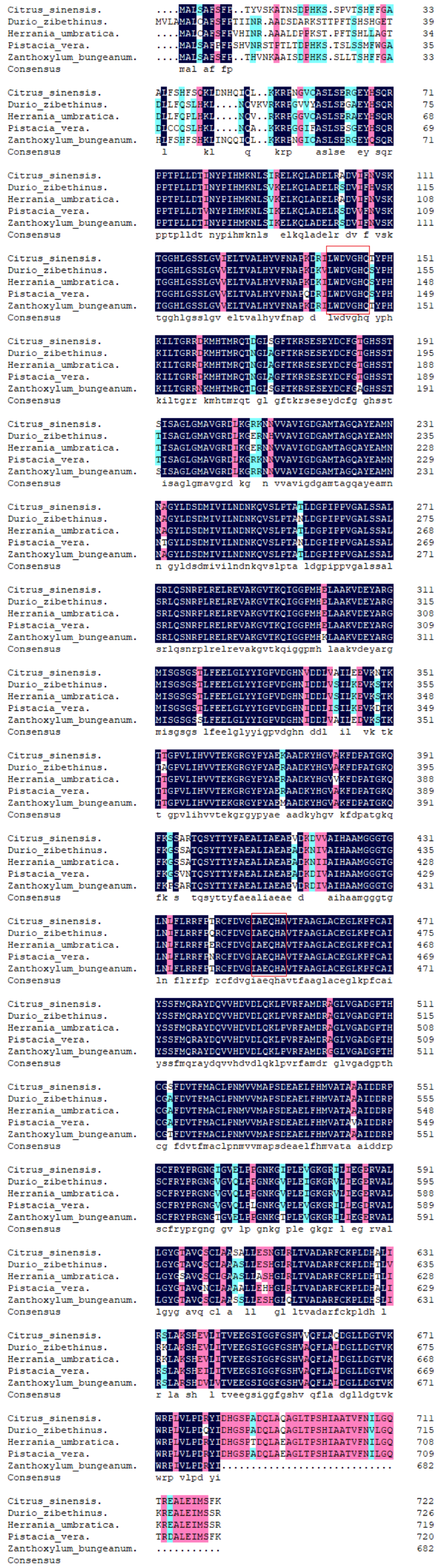

Supplement: Supplementary file 1 [file foods-11-01746-s001.zip › Figure_S2.pdf]
