# Supplementary material for: Molecular Cloning and Functional Analysis of DXS and FPS Genes from Zanthoxylum bungeanum Maxim
Source: Foods. 2022 Jun 14;11(12):1746. doi: 10.3390/foods11121746 (PMC9223008; doi:10.3390/foods11121746)
Supplement: Supplementary file 1 [file foods-11-01746-s001.zip › Figure_S3.pdf]

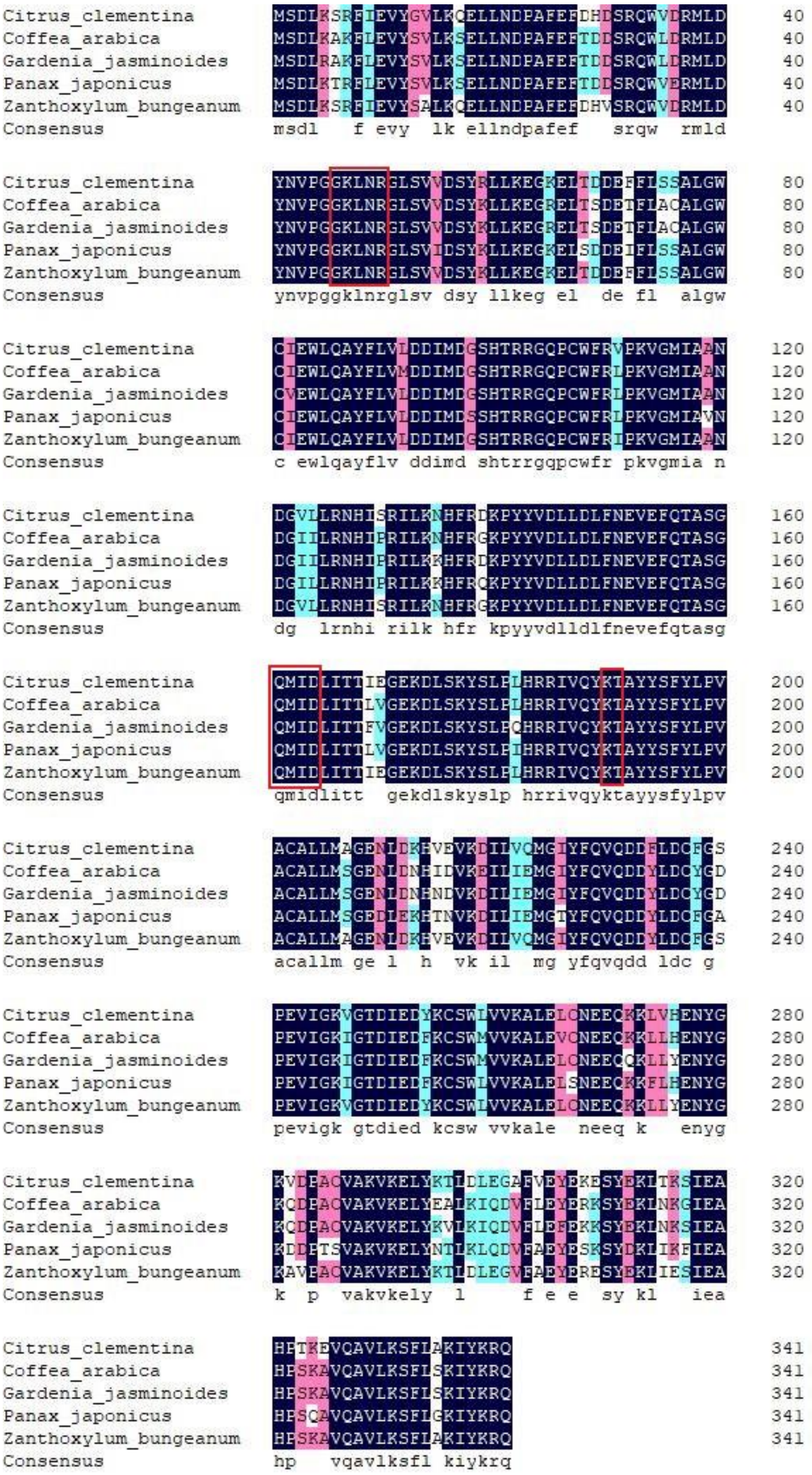
Citrus_clementina MSDLKSRFIEVYGVLKQELLNDPAFEFDHDSRQWVDRMLD 40
Coffea_arabica MSDLKAKFLEVYSVLKSELLNDPAFEFTDDSRQWLDRMLD 40
Gardenia_jasminoides MSDLRAKFLEVYSVLKSELLNDPAFEFTDDSRQWLDRMLD 40
Panax_japonicus MSDLKTRFLEVYSVLKSELLNDPAFEFTDDSRQWVERMLD 40
Zanthoxylum_bungeanum MSDLKSRFIEVYSALKQELLNDPAFEFDHVSRQWVDRMLD 40
Consensus msdl f evy lk ellndpafef srqw rmld
Citrus_clementina YNVPGGKLNRGLSVVDSYRLLKEGKELTDDEFFLSSALGW 80
Coffea_arabica YNVPGGKLNRGLSVVDSYKLLKEGRELTSDETFLACALGW 80
Gardenia_jasminoides YNVPGGKLNRGLSVVDSYKLLKEGRELTSDETFLACALGW 80
Panax_japonicus YNVPGGKLNRGLSVIDSYKLLKEGKELSDDEIFLSSALGW 80
Zanthoxylum_bungeanum YNVPGGKLNRGLSVVDSYKLLKEGKELTDDEFFLSSALGW 80
Consensus ynvpggklnrglsv dsy llkeg el de fl algw
Citrus_clementina CIEWLQAYFLVLDDIMDGSHTRRGQPCWFRVPKVGMIAAN 120
Coffea_arabica CIEWLQAYFLVMDDIMDGSHTRRGQPCWFRLPKVGMIAAN 120
Gardenia_jasminoides CVEWLQAYFLVLDDIMDGSHTRRGQPCWFRLPKVGMIAAN 120
Panax_japonicus CIEWLQAYFLVLDDIMDSSHTRRGQPCWFRLPKVGMIAVN 120
Zanthoxylum_bungeanum CIEWLQAYFLVLDDIMDGSHTRRGQPCWFRIPKVGMIAAN 120
Consensus c ewlqayflv ddimd shtrrgqpcwfr pkvgmia n
Citrus_clementina DGVLLRNHISRILKNHFRDKPYYVDLLDLFNEVEFQTASG 160
Coffea_arabica DGIILRNHIPRILKNHFRGKPYYVDLLDLFNEVEFQTASG 160
Gardenia_jasminoides DGIILRNHIPRILKKHFRDKPYYVDLLDLFNEVEFQTASG 160
Panax_japonicus DGILLRNHIPRILKKHFRQKPYYVDLLDLFNEVEFQTASG 160
Zanthoxylum_bungeanum DGVLLRNHISRILKNHFRGKPYYVDLLDLFNEVEFQTASG 160
Consensus dg lrnhi rilk hfr kpyyvdlldlfnevefqtasg
Citrus_clementina QMIDLITTIEGEKDLSKYSLPLHRRIVQYKTAYYSFYLPV 200
Coffea_arabica QMIDLITTLVGEKDLSKYSLPLHRRIVQYKTAYYSFYLPV 200
Gardenia_jasminoides QMIDLITTFVGEKDLSKYSLPQHRRIVQYKTAYYSFYLPV 200
Panax_japonicus QMIDLITTLVGEKDLSKYSLPIHRRIVQYKTAYYSFYLPV 200
Zanthoxylum_bungeanum QMIDLITTIEGEKDLSKYSLPLHRRIVQYKTAYYSFYLPV 200
Consensus qmidlitt gekdlskyslp hrrivqyktayysfylpv
Citrus_clementina ACALLMAGENIDKEVEVKDILVQMGIYFQVQDDFLDCFGS 240
Coffea_arabica ACALLMSGENIDNHIDVKEILIEMGIYFQVQDDYLDCYGD 240
Gardenia_jasminoides ACALLMSGENIDNENDVKDILIEMGIYFQVQDDYLDCYGD 240
Panax_japonicus ACALLMSGEDIEKHTNVKDILIEMGTYFQVQDDYLDCFGA 240
Zanthoxylum_bungeanum ACALLMAGENIDKEVEVKDILVQMGIYFQVQDDYLDCFGS 240
Consensus acallm ge l h vk il mg yfqvqdd ldc g
Citrus_clementina PEVIGKVGTDIEDYKCSWLVVKALELCNEEQKKLVHENYG 280
Coffea_arabica PEVIGKIGTDIEDFKCSWMVVKALEVCNEEQKKLLHENYG 280
Gardenia_jasminoides PEVIGKIGTDIEDFKCSWMVVKALELCNEEQQKLLYENYG 280
Panax_japonicus PEVIGKIGTDIEDFKCSWLVVKALELSNEEQKKFLHENYG 280
Zanthoxylum_bungeanum PEVIGKVGTDIEDYKCSWLVVKALELCNEEQKKLLYENYG 280
Consensus pevigk gtdied kcsw vvkale neeq k enyg
Citrus_clementina KVDPACVAKVKELYKTLDLEGAFVEYEKESYEKLTKSIEA 320
Coffea_arabica KQDPACVAKVKELYEALKIQDVFLEYERKSYEKLNKGIEA 320
Gardenia_jasminoides KQDPACVAKVKELYKVLKIQDVFLEFEKKSYEKLNKSIEA 320
Panax_japonicus KDDPTSVAKVKELYNTLKLQDVFAEYESKSYDKLIKFIEA 320
Zanthoxylum_bungeanum KAVPACVAKVKELYKTLDLEGVFAEYERESYEKLIESIEA 320
Consensus k p vakvkely l f e e sy kl iea
Citrus_clementina HPTKEVQAVLKSFLAKIYKRQ 341
Coffea_arabica HPSKAVQAVLKSFLSKIYKRQ 341
Gardenia_jasminoides HPSKAVQAVLKSFLSKIYKRQ 341
Panax_japonicus HPSQAVQAVLKSFLGKIYKRQ 341
Zanthoxylum_bungeanum HPSKAVQAVLKSFLAKIYKRQ 341
Consensus hp vqavlksfl kiykrq

Figure S3. Deduced amino acid sequences of the conserved regions of ZbFPS. The conserved domains are boxed. Homology level was highlighted by shading in color: black for 100%, pink for ⩾ 75% and blue for ⩾ 50% identity.
