# Supplementary material for: Molecular Cloning and Functional Analysis of DXS and FPS Genes from Zanthoxylum bungeanum Maxim
Source: Foods. 2022 Jun 14;11(12):1746. doi: 10.3390/foods11121746 (PMC9223008; doi:10.3390/foods11121746)
Supplement: Supplementary file 1 [file foods-11-01746-s001.zip › Figure_S4.pdf]

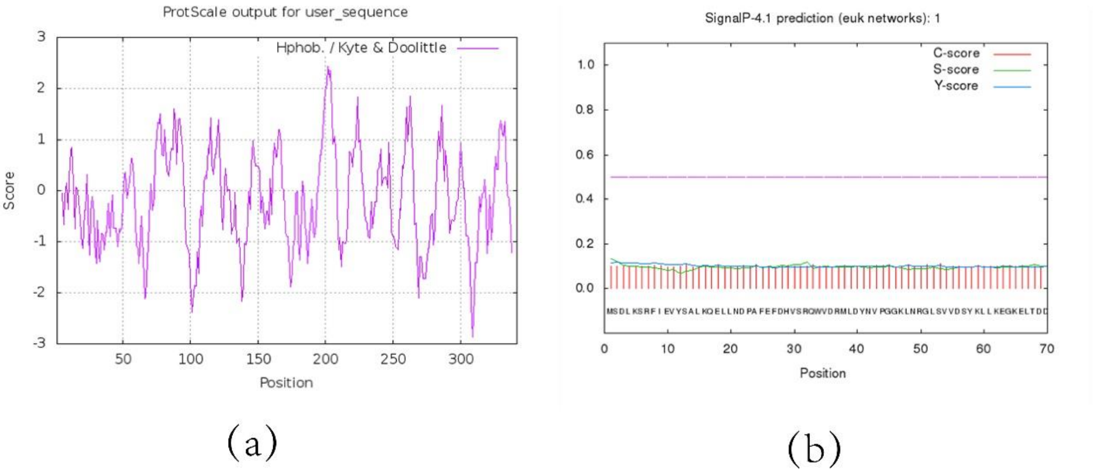

Figure S4. Analysis of protein sequence traits. Hydrophobicity and hydrophilicity prediction of ZbFPS (a). Signal peptide analysis result of ZbFPS (b). C-score: possibility of cleavage site; S-score: possibility of amino acid being signal peptide; Y-score: derivative of binding C-score and S-score.
