# Supplementary material for: Molecular Cloning and Functional Analysis of DXS and FPS Genes from Zanthoxylum bungeanum Maxim
Source: Foods. 2022 Jun 14;11(12):1746. doi: 10.3390/foods11121746 (PMC9223008; doi:10.3390/foods11121746)
Supplement: Supplementary file 1 [file foods-11-01746-s001.zip › Table_S1.pdf]

Table S1. Primers used in the experiment

Primers	Sequence (5′-3′)	Description
FPS-pET28a-F	TGGGTCGCGGATCCGAATTCAGTGATCTGAAGTCGAGATT	Prokaryotic expression for *FPS*
FPS-pET28a-R	TCGAGTGCGGCCGCAAGCTTGTTACTTCTGTCTCTTGTATATCTTAGC	Prokaryotic expression for *FPS*
FPS-pBI121-F	AGAACACGGGGGACTCTAGACAGTGATCTGAAGTCGAGATT	Transient expression for *FPS*
FPS-pBI121-R	GACTGACCACCCGGGGATCCTTACTTCTGTCTCTTGTATATCTTAGC	Transient expression for *FPS*
DXS-pET28a-F	TGGGTCGCGGATCCGAATTCGCTCTCTCTGCATTCTCATTTCCAAC	Prokaryotic expression for *DXS*
DXS-pET28a-R	TCGAGTGCGGCCGCAAGCTTGTTAGTTTTTGAATGACATTATCTCGAGAGCC	Prokaryotic expression for *DXS*
DXS-pBI121-F	AGAACACGGGGGACTCTAGACGCTCTCTCTGCATTCTCA	Transient expression for *DXS*
DXS-pBI121-R	GACTGACCACCCGGGGATCC TTAGTTTTTGAATGACATTATCTCG	Transient expression for *DXS*
DXS-F	TCAATCTCAAAGACCACCAACACCTC	RT-qPCR of *DXS*
DXS-R	GACATCCGACCGCAACTCATCTG	RT-qPCR of *DXS*
FPS-F	GCCACTTCATCGTCGCATTGTTC	RT-qPCR of *FPS*
FPS-R	TGTCAAGATTCTCGCCTGCCATAAG	RT-qPCR of *FPS*
UBQ-F	TCGAAGATGGCCGTACATTG	RT-qPCR of *UBQ*
UBQ-R	TCCTCTAAGCCTCAGCACCA	RT-qPCR of *UBQ*
UBA-F	GACTTAGGGGAGGGATTATTGAG	RT-qPCR of *UBA*
UBA-R	TTCTTCTTCCGACAGTTTACAGC	RT-qPCR of *UBA*
