# Supplementary material for: Molecular Cloning and Functional Analysis of DXS and FPS Genes from Zanthoxylum bungeanum Maxim
Source: Foods. 2022 Jun 14;11(12):1746. doi: 10.3390/foods11121746 (PMC9223008; doi:10.3390/foods11121746)
Supplement: Supplementary file 1 [file foods-11-01746-s001.zip › Table_S2.pdf]

Table S2. Sequence information for ZbFPS.

Gene name	Amino acid sequence (aa)	Molecular weight (kDa)	PI	Formula	Instability index	Aliphatic index
ZbFPS	342	39.472	5.53	$C_{1797}H_{2789}N_{455}O_{516}S_{14}$	37.69	97.16
