# Supplementary material for: Molecular Cloning and Functional Analysis of DXS and FPS Genes from Zanthoxylum bungeanum Maxim
Source: Foods. 2022 Jun 14;11(12):1746. doi: 10.3390/foods11121746 (PMC9223008; doi:10.3390/foods11121746)
Supplement: Supplementary file 1 [file foods-11-01746-s001.zip › Table_S3.pdf]

Table S3. Sequence identity, GMQE, and QMEAN for ZbDXS and ZbFPS

Gene name	Seq Identity	GMQE	QMEAN
ZbDXS	41.34%	0.63	-3.04
ZbFPS	82.06%	0.96	-0.06
