# Supplementary material for: Molecular Cloning and Functional Analysis of DXS and FPS Genes from Zanthoxylum bungeanum Maxim
Source: Foods. 2022 Jun 14;11(12):1746. doi: 10.3390/foods11121746 (PMC9223008; doi:10.3390/foods11121746)
Supplement: Supplementary file 1 [file foods-11-01746-s001.zip › Table_S4.pdf]

Table S4. Relative expression levels of *ZbFPS* and *ZbDXS* in different tissues.

Different Tissues	Genes	
	ZbDXS	*ZbFPS*
Roots	1	1
Stems	4.9807±0.98	0.5053±0.20
Leaves	9.7358±1.07	2.1202±0.67
Flowers	2.8984±0.70	0.7868±0.17
Fruits	39.9223±2.96	4.2896±0.99

Note: Taking the gene expression in roots as a reference. The relative mRNA abundance was calculated using the formula: $2^{-\Delta\Delta Ct}$. Data are based on a statistical analysis of the means of three biological replications (n=3).
