# Supplementary figures and images for: Molecular Cloning and Functional Analysis of DXS and FPS Genes from Zanthoxylum bungeanum Maxim
Source: Foods. 2022 Jun 14;11(12):1746. doi: 10.3390/foods11121746 (PMC9223008; doi:10.3390/foods11121746)

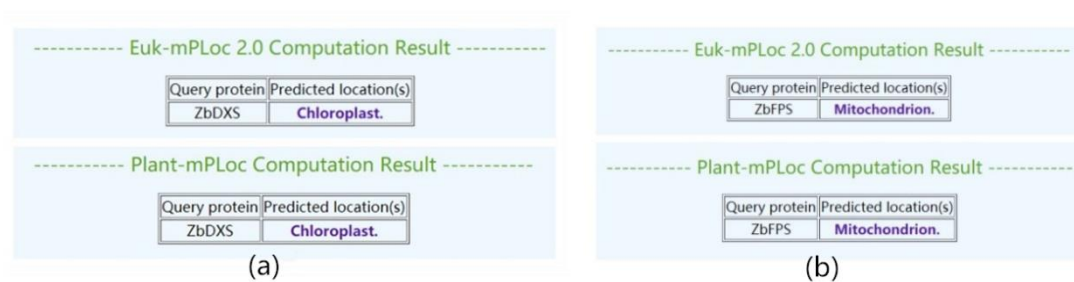

**Figure S5.** Predicting subcellular localization of ZbDXS (a) and ZbFPS (b).

Supplement: Supplementary file 1 [file foods-11-01746-s001.zip › Figure_S5.pdf]
